# Exploring the impact of artificial intelligence on teaching and learning in higher education

**DOI:** 10.1186/s41039-017-0062-8

**Published:** 2017-11-23

**Authors:** Stefan A. D. Popenici, Sharon Kerr

**Affiliations:** 1 Office of Learning and Teaching, Charles Darwin University, Casuarina Campus, Orange 1.2.15, Ellengowan Drive, Darwin, Northern Territory 0909 Australia; 2 Global Access Project, HECG Higher Education Consulting Group, Level 11 10 Bridge Street, Sydney, New South Wales 2000 Australia

**Keywords:** Higher education, Artificial intelligence, Teacherbots, Augmentation, Machine learning, Teaching, Graduate attributes

## Abstract

This paper explores the phenomena of the emergence of the use of artificial intelligence in teaching and learning in higher education. It investigates educational implications of emerging technologies on the way students learn and how institutions teach and evolve. Recent technological advancements and the increasing speed of adopting new technologies in higher education are explored in order to predict the future nature of higher education in a world where artificial intelligence is part of the fabric of our universities. We pinpoint some challenges for institutions of higher education and student learning in the adoption of these technologies for teaching, learning, student support, and administration and explore further directions for research.

## Introduction

The future of higher education is intrinsically linked with developments on new technologies and computing capacities of the new intelligent machines. In this field, advances in artificial intelligence open to new possibilities and challenges for teaching and learning in higher education, with the potential to fundamentally change governance and the internal architecture of institutions of higher education. With answers to the question of ‘what is artificial intelligence’ shaped by philosophical positions taken since Aristotle, there is little agreement on an ultimate definition.

In 1950s, Alan Turing proposed a solution to the question of when a system designed by a human is ‘intelligent.’ Turing proposed the imitation game, a test that involves the capacity of a human listener to make the distinction of a conversation with a machine or another human; if this distinction is not detected, we can admit that we have an intelligent system, or artificial intelligence (AI). It is worth remembering that the focus on AI solutions goes back to 1950s; in 1956 John McCarthy offered one of the first and most influential definitions: “The study [of artificial intelligence] is to proceed on the basis of the conjecture that every aspect of learning or any other feature of intelligence can in principle be so precisely described that a machine can be made to simulate it.” (Russell and Norvig 2010).

Since 1956, we find various theoretical understandings of artificial intelligence that are influenced by chemistry, biology, linguistics, mathematics, and the advancements of AI solutions. However, the variety of definitions and understandings remains widely disputed. Most approaches focus on limited perspectives on cognition or simply ignore the political, psychological, and philosophical aspects of the concept of intelligence. For the purpose of our analysis of the impact of artificial intelligence in teaching and learning in higher education, we propose a basic definition informed by the literature review of some previous definitions on this field. Thus, we can define artificial intelligence (AI) as computing systems that are able to engage in human-like processes such as learning, adapting, synthesizing, self-correction and use of data for complex processing tasks.

Artificial intelligence is currently progressing at an accelerated pace, and this already impacts on the profound nature of services within higher education. For example, universities already use an incipient form of artificial intelligence, IBM’s supercomputer Watson. This solution provides student advice for Deakin University in Australia at any time of day throughout 365 days of the year (Deakin University 2014). Even if it is based on algorithms suitable to fulfill repetitive and relatively predictable tasks, Watson’s use is an example of the future impact of AI on the administrative workforce profile in higher education. This is changing the structure for the quality of services, the dynamic of time within the university, and the structure of its workforce. A supercomputer able to provide bespoke feedback at any hour is reducing the need to employ the same number of administrative staff previously serving this function. In this context, it is also important to note that ‘machine learning’ is a promising field of artificial intelligence. While some AI solutions remain dependent on programming, some have an inbuilt capacity to learn patterns and make predictions. An example is AlphaGo—a software developed by DeepMind, the AI branch of Google’s—that was able to defeat the world’s best player at Go, a very complex board game (Gibney 2017). We define ‘machine learning’ as a subfield of artificial intelligence that includes software able to recognize patterns, make predictions, and apply the newly discovered patterns to situations that were not included or covered by their initial design.

## Results and discussion

As AI solutions have the potential to structurally change university administrative services, the realm of teaching and learning in higher education presents a very different set of challenges. Artificial intelligence solutions relate to tasks that can be automated, but cannot be yet envisaged as a solution for more complex tasks of higher learning. The difficulty of supercomputers to detect irony, sarcasm, and humor is marked by various attempts that are reduced to superficial solutions based on algorithms that can search factors such as a repetitive use of punctuations marks, use of capital letters or key phrases (Tsur et al. 2010). There is a new hype about possibilities of AI in education, but we have reasons to stay aware of the real limits of AI algorithmic solutions in complex endeavors of learning in higher education.

For example, we can remember that the enthusiastic and unquestioned trust in the AI capabilities of a revolutionary new car led on May 2016 to the death of the driver, when the car set on ‘autopilot’ went underneath a tractor-trailer that was not detected by the software (Reuters/ABC 2016). There is also the story of Microsoft’s embarrassing mistake to trust the AI-powered bot named Tay to go unsupervised on Twitter. Confident on the bot capacity to operate independently, Microsoft discovered that Tay turned fast into a racist, bigoted, and hate-spewing account. ‘Tay’ had to be shut down by Microsoft after only 16 h of work. For example, Tay answered the question “Are you a racist?” with a disturbing “because ur mexican”. A Microsoft spokesperson explained that: “The AI chatbot Tay is a machine learning project, designed for human engagement. It is as much a social and cultural experiment, as it is technical. Unfortunately, within the first 24 hours of coming online, we became aware of a coordinated effort by some users to abuse Tay’s commenting skills to have Tay respond in inappropriate ways. As a result, we have taken Tay offline and are making adjustments.” (Perez 2016).

There is consistent evidence—some presented in this paper—that AI solutions open a new horizon of possibilities for teaching and learning in higher education. However, it is important to admit the current limits of technology and admit that AI is not (yet) ready to replace teachers, but is presenting the real possibility to augment them. We are now seeing computing algorithms impacting on the most mundane aspects of daily life, from individuals’ credit scores to employability. Higher education is placed at the center of this profound change, which brings with it both extraordinary opportunities and risks. This important crossroad requires careful consideration and analysis from an academic perspective, especially as we can find tendencies to look at technological progress as a solution or replacement for sound pedagogical solutions or good teaching. The real potential of technology in higher education is—when properly used—to extend human capabilities and possibilities of teaching, learning, and research. The purpose of this paper is to kindle scholarly discussions on the evolving field of artificial intelligence in higher education. This stays aligned with some of the most ambitious research agendas in the field, such as the “National Artificial Intelligence Research and Development Strategic Plan,” released by the US President Barack Obama in October 2016. The Report states that “the walls between humans and AI systems are slowly beginning to erode, with AI systems augmenting and enhancing human capabilities. Fundamental research is needed to develop effective methods for human-AI interaction and collaboration” (U.S. National Science and Technology Council 2016).

As we note that significant advances in machine learning and artificial intelligence open new possibilities and challenges for higher education, it is important to observe that education is eminently a human-centric endeavor, not a technology centric solution. Despite rapid advancements in AI, the idea that we can solely rely on technology is a dangerous path, and it is important to maintain focus on the idea that humans should identify problems, critique, identify risks, and ask important questions that can start from issues such as privacy, power structures, and control to the requirement of nurturing creativity and leaving an open door to serendipity and unexpected paths in teaching and learning. The hype on AI can lead to an unquestioned panacea that can leave many who are on their path to higher learning under the wheels of reality, such as that tragic event of the driver led under a truck by what was considered to be a matchless software. Maintaining academic skepticism on this issue is especially important in education, as this is an act that can be reduced to information delivery and recollection; we need to maintain its aim to build educated minds and responsible citizens that are attached to general values of humanism.

The role of technology in higher learning is to enhance human thinking and to augment the educational process, not to reduce it to a set of procedures for content delivery, control, and assessment. With the rise of AI solutions, it is increasingly important for educational institutions to stay alert and see if the power of control over hidden algorithms that run them is not monopolized by tech-lords. Frank Pasquale notes in his seminal book ‘The Black Box Society’ that “Decisions that used to be based on human reflection are now made automatically. Software encodes thousands of rules and instructions computed in a fraction of a second” (Pasquale 2015). Pasquale is revealing in his book that we do not only have a quasi-concentrated and powerful monopoly over these solutions, but also an intentional lack of transparency on algorithms and how they are used. This is presented casually as a normal state of facts, the natural arrangements of Internet era, but it translates to highly dangerous levels of unquestioned power. Those who control algorithms that run AI solutions have now unprecedented influence over people and every sector of a contemporary society. The internal architecture of the mega-corporations such as Facebook or Google is not following a democratic model, but those of benevolent dictators who know what is best and decide with no consultation with their internal or external subjects. The monopoly and the strong control over sources of information, stifling critique and silencing de facto through *invisibilisation* views that are not aligned with interest and narratives promoted by techlords’ interests stand in direct opposition with higher learning. Universities have a role if they encourage dissent and open possibilities revealed by it. Higher learning is withering when the freedom of thinking and inquiry is suppressed in any form, as manipulations and the limitation of knowledge distorts and cancel in-depth understandings and the advancement of knowledge. If we reach a point where the agenda of universities is set by a handful of techlords, as well as the control over their information and the ethos of universities, higher education is looking ahead a very different age. The set of risks is too important to be overlooked and not explored with courage and careful analysis.

At the same time, the rapid advancements of AI are doubled by the effort of defunded universities to find economic solutions to balance depleted budgets. AI already presents the capability to replace a large number of administrative staff and teaching assistants in higher education. It is therefore important to explore the effects of these factors on learning in higher education, especially in the context of an increasing demand for initiative, creativity, and ‘entrepreneurial spirit’ for graduates. This paper opens an inquiry into the influence of artificial intelligence (AI) on teaching and learning and higher education. It also operates as an exploratory analysis of literature and recent studies on how AI can change not only how students learn in universities, but also on the entire architecture of higher education.

### The rise of artificial intelligence and augmentation in higher education

The introduction and adoption of new technologies in learning and teaching has rapidly evolved over the past 30 years. Looking through the current lens, it is easy to forget the debates that have raged in our institutions over students being allowed to use what are now regarded as rudimentary technologies. In a longitudinal study of accommodations for students with a disability conducted between 1993 and 2005 in the USA, authors remind us of how contentious the debate was surrounding the use of the calculators and spell check programs for students with a disability none-the-less the general student body (Lazarus et al. 2008). Assistive technologies—such as text to speech, speech to text, zoom capacity, predictive text, spell checkers, and search engines—are just some examples of technologies initially designed to assist people with a disability. The use of these technological solutions was later expanded, and we find them now as generic features in all personal computers, handheld devices or wearable devices. These technologies now augment the learning interactions of all students globally, enhancing possibilities opened for teaching and design of educational experiences.

Moreover, artificial intelligence (AI) is now enhancing tools and instruments used day by day in cities and campuses around the world. From Internet search engines, smartphone features and apps, to public transport and household appliances. For example, the complex set of algorithms and software that power iPhone’s Siri is a typical example of artificial intelligence solutions that became part of everyday experiences (Bostrom and Yudkowsky 2011; Luckin 2017). Even if Apple’s Siri is labeled as a low complexity AI solution or simply a voice controlled computer interface, it is important to remember that it started as an artificial intelligence project funded in the USA by the Defense Advanced Research Projects Agency (DARPA) since 2001. This project was turned a year later into a company that was acquired by Apple, which integrated the application in its iPhone operation system in 2007. Google is using AI for its search engines and maps, and all new cars use AI from engine to breaks and navigation. Self-driving technology is already advanced, and some major companies are making this a top priority for development, such as Tesla, Volvo, Mercedes, and Google (Hillier et al. 2015) and trials on public roads in Australia commenced in 2015. Remarkably, a mining corporation is already taking advantage of self-driving technologies, now using self-driving trucks for two major exploitations in Western Australia (Diss 2015).

Personalized solutions are also closer than we imagined: ‘new scientist’ presented at the end of 2015 the initiative of Talkspace and IBM’s Watson to use artificial intelligence in psychotherapy (Rutkin 2015). This seems to be a major step towards changing the complex endeavor of education with AI. In fact, Nick Bostrom, Director of the Future of Humanity Institute at the UK’s Oxford University, observed since 2006 that artificial intelligence is now an integral part of our daily life: “A lot of cutting edge AI has filtered into general applications, often without being called AI because once something becomes useful enough and common enough it’s not labelled AI anymore” (Bostrom 2006). Again, very few people identify today Siri as a typical example of artificial intelligence and more as an algorithm-based personal assistant that is part of everyday life experiences. Given their increasing role within the global digital infrastructure, this also begs the question as to how algorithms are conceived of as we prepare ourselves for a range of different possible futures.

Students are placed now at the forefront of a vast array of possibilities and challenges for learning and teaching in higher education. Solutions for human-AI interaction and collaboration are already available to help people with disabilities. They can inspire educators to apply them in education to augment learners and teachers for a more engaging process. Carl Mitcham describes in his Encyclopedia of Science, Technology and Ethics a cyborg as “a crossbreed of a human and a machine” (Mitcham 2005). The idea of cyborgs is not as far away as we may imagine, as the possibilities to combine human capacities with new technologies are already being used and developed at an accelerated pace. For example, Hugh Herr, who is directing the Biomechatronics group at the MIT Media Lab and works with the Harvard–MIT Division of Health Sciences and Technology, recently observed in an interview for ‘new scientist’ that “…disability will end, I’d say, by the end of this century. And I think that’s a very conservative statement. At the rate technology is progressing, most disability will be gone in 50 years” (De Lange 2015, p. 25). This company is producing technologically advanced prosthetics and exoskeletons, pioneering bionic technology for people with or *without* a disability. He notes that his research group developed an interface that “uses biology to close the loop between human and machine […] Imagine a world where our physicality doesn’t decrease as we age” (De Lange 2015, p. 24). Complex computing systems using machine learning algorithms can serve people with all types of abilities and engage to a certain degree in human-like processes and complex processing tasks that can be employed in teaching and learning. This opens to a new era for institutions of higher education.

This type of human-machine interface presents the immediate potential to change the way we learn, memorize, access, and create information. The question of how long it will take to use this type of interface to enhance human memory and cognition is one which we are currently unable to answer. It may turn to reality beyond the end of this century, as the MIT scholar suggests or much sooner when we consider the pace of change in the technologies used in teaching and learning since 2007 when the first iPhone was launched. Since then, not only has the iPhone integrated breakthrough technologies that seemed impossible just a few years ago to how we access and use information (such as fingerprint identification and the ‘intelligent’ Siri assistant), but this technology has introduced a significant cultural shift that impacts on our everyday lives. Either way, if we shift the focus of ‘cyborgs’ from science-fiction to the idea of computer augmented capacity for teachers and students alike, it is not unrealistic to consider that cyborgs—or ‘crossbreeds’ of human and machines—will soon be a reality in teaching and research in universities of the near future.

The impact of artificial intelligence is already visible in the world economy and has captured the attention of many analysts. The largest investment ever made by Google in the European Union is the acquisition in 2014 of DeepMind technologies, with $400 million. DeepMind Technologies, now named Google DeepMind, is a London-based artificial intelligence startup specialized in machine learning and advanced algorithms. Notably, Google also made significant investments in the German Research Centre for Artificial Intelligence (DFKI GmbH), which is, according to their website, “the biggest research center worldwide in the area of Artificial Intelligence and its application, in terms of number of employees and the volume of external funds” (DFKI 2015). Tech giants like Apple, Google, Microsoft, and Facebook currently compete in the field of artificial intelligence and are investing heavily in new applications and research. Google announced in December 2015 that the company’s quantum computer called D-Wave 2X will be used for complex operations of AI, generically referred to as optimization problems (Neven 2015). This new machine is 100 million times faster than any other contemporary computers, a serious leap ahead for AI, considered by Google researchers as a significant breakthrough: “We hope it helps researchers construct more efficient and more accurate models for everything from speech recognition, to web search, to protein folding” (Neven 2013).

This wave of interest and investments in artificial intelligence will soon impact on universities. Most likely, financial pressures related to the large numbers of students currently undertaking higher education driven by the goal of democratization of higher education, and the international student market will stand as a compelling reason to seek out AI solutions. The ‘outsourcing’ of the academic workforce, in terms of numbers of academics employed and tenured positions, is now open to a massive takeover by intelligent machines (Grove 2015). ‘Massification’ of higher education and the political call to cut public funding for universities translates into a real need to cut costs. With research still being the main source of funds and prestige in international rankings, the MOOC hype unveiled the tempting solution for many university administrators to cut costs by reducing expensive academic teaching staff. This shift is currently being aggressively pursued in Australian universities, with a constant shift towards casual and short-term contracts; in a study conducted by L.H. Martin Institute it is documented that “…there is an escalating trend in the number and percentage of academic staff on contingent appointments, and a declining trend in the percentage of academic staff with continuing appointments who undertake both teaching and research” (Andrews et al. 2016). In the UK, we find various initiatives following the same trend, such as that of University of Warwick, which created a new department to employ all casual teaching staff to outsource teaching. This new department was established to function in a way “similar to another subsidiary used to pay cleaners and catering staff, suitable to serve the University of Warwick and also sell teaching and assessment services to other institutions” (Gallagher 2015).

As examples presented in previous page show, the “crossbreed” of the human brain and a machine is already possible, and this will essentially challenge teachers to find new dimensions, functions, and radically new pedagogies for a different context for learning and teaching. For example, brain-computer interfaces (BCIs), that captured the imagination of researchers across the world, are currently recording significant advances. Using brain signals with various recording and analysis methods, along with innovative technological approaches for new computing systems, specialists in the field now provide feasible solutions to remotely control software with a brain-computer interface (Andrea et al. 2015). BCIs are now able to capture and decode brain activity to enable communication and control by individuals with motor function disabilities (Wolpaw and Wolpaw 2012). Kübler et al. observe that at this point “studies have demonstrated fast and reliable control of brain-computer interfaces (BCIs) by healthy subjects and individuals with neurodegenerative disease alike” (Kübler et al. 2015). The concept of humanity and the possibilities of humans stand currently to be redefined by technology with unprecedented speed: technology is quickly expanding the potential to use AI functions to enhance our skills and abilities. As Andreas Schleicher observed, “Innovation in education is not just a matter of putting more technology into more classrooms; it is about changing approaches to teaching so that students acquire the skills they need to thrive in competitive global economies” (Schleicher 2015).

### Past lessons, possibilities, and challenges of AI solutions

Widening participation in higher education and the continuous increase in the number of students, class sizes, staff costs, and broader financial pressures on universities makes the use of technology or teacherbots a very attractive solution. This became evident when massive open online courses (MOOCs) enlightened the imagination of many university administrators. The understanding of “open courses” is that no entry requirements or fees were required, and online students could enroll and participate from any country in the world with internet access. Both of these factors enabled universities to market globally for students, resulting in massive enrolment numbers. The promise was generous, but it soon became evident that one of the problems created for teachers was their human capacity to actively engage with massive numbers of diverse students studying globally from different time zones, at different rates of progress and with different frames of reference and foundational skills for the course that they are studying. Assisting students in large classes to progress effectively through their learning experience to achieve desired outcomes, conduct assessments, and provide constructive personalized feedback remained unsolved issues. Sian Bayne makes the observation in *Teacherbot: Interventions in Automated Teaching*, that the current perspective of using automated methods in teaching “are driven by a productivity-oriented solutionism,” not by pedagogical or charitable reasoning, so we need to re-explore a humanistic perspective for mass education to replace the “cold technocratic imperative” (Bayne 2015). Bayne speaks from the experience of meeting the need created by the development and delivery of a massive open online course by the University of Edinburgh. This course had approximately 90,000 students from 200 countries enrolled.

The lesson of MOOCs is important and deserves attention. Popenici and Kerr observed that MOOCs were first used in 2008 and since then: “…we have been hearing the promise of a *tsunami of change* that is coming over higher education. It is not uncommon with a tsunami to see people enticed by the retreat of the waters going to collect shells, thinking that this is the change that is upon them. Tragically, the real change is going to come in the form of a massive wave under which they will perish as they play on the shores. Similarly, we need to take care that we are not deluded to confuse MOOCs, which are figuratively just shells on the seabed, with the massive wall of real change coming our way” (Popenici and Kerr 2013). It is becoming clear in 2016 that MOOCs remain just a different kind of online course, interesting and useful, but not really aimed at or capable of changing the structure and function of universities. Research and data on this topic reflect the failure of MOOCs to deliver on their proponents’ promises. More importantly, the unreserved and irrational hype that surrounded MOOCs is a when decision-makers in academia decided to ignore all key principles—such as evidence-based arguments or academic skepticism—and embrace a fad sold by Silicon Valley venture capitalists with no interests in learning other than financial profits. As noted in a recent book chapter “this reckless shift impacts on the sustainability of higher learning in particular and of higher education by and large” (Popenici 2015).

There are solid arguments—some cited above in this paper—to state that it is more realistic to consider the impact of machine learning in higher education as the real wave of change. In effect, lessons of the past show why it is so important to avoid the same mistakes revealed by the past fads or to succumb to a convenient complacency that is serving only the agenda of companies that are in search of new (or bigger) markets. Online learning proved very often the potential to successfully help institutions of higher education reach some of the most ambitious goals in learning, teaching, and research. However, the lesson of MOOCs is also that a limited focus on one technology solution without sufficient evidence-based arguments can become a distraction for education and a perilous pathway for the financial sustainability of these institutions.

Higher education is now taking its first steps into the unchartered territory of the possibilities opened by AI in teaching, learning, and higher education organization and governance. Implications and possibilities of these technological advances can already be seen. By way of example, recent advancements in non-invasive brain-computer interfaces and artificial intelligence are opening new possibilities to rethink the role of the teacher, or make steps towards the replacement of teachers with teacher-robots, virtual “teacherbots” (Bayne 2015; Botrel et al. 2015). Providing affordable solutions to use brain computer interface (BCI) devices capable to measure when a student is fully focused on the content and learning tasks (Chen et al. 2015; González et al. 2015) is already possible, and super-computers, such as IBM’s Watson, can provide an automated teacher presence for the entire duration of a course. The possibility to communicate and command computers through thought and wider applications of AI in teaching and learning represents the real technological revolution that will dramatically change the structure of higher education across the world. Personalized learning with a teacherbot, or ‘cloud-lecturer’, can be adopted for blended delivery courses or fully online courses. Teacherbots—computing solutions for the administrative part of teaching, dealing mainly with content delivery, basic and administrative feedback and supervision—are already presenting as a disruptive alternative to traditional teaching assistants. An example is offered by the course offered by Professor Ashok Goel on knowledge-based artificial intelligence (KBAI) in the online Master in Computer Sciences program, at Georgia Tech in the USA. The teaching assistant was so valued by students that one wanted to nominate her to the outstanding TA award. This TA managed to meet the highest expectations of students. The surprise at the end of the course was to find out that Jill Watson was not a real person, but a teacherbot, a virtual teaching assistant was based on the IBM’s Watson platform (Maderer 2016).

This enlightened the imaginations of many, reaching international news across the world and respected media outlets such as *The New Your Times* or *The Washington Post*. However, we must be careful when we see the temptation to equate education with solutions provided by algorithms. There are widespread implications for the advancement of AI to the point where a computer can serve as a personalized tutor able to guide and manage students’ learning and engagement. This opens to the worrying possibility to see a superficial, but profitable, approach where teaching is replaced by AI automated solutions. Especially as we are at a point where we need to find a new pedagogical philosophy that can help students achieve the set of skills required in the twenty-first century for a balanced civic, economic, and social life. We have a new world that is based on uncertainty and challenges that change at a rapid pace, and all this requires creativity, flexibility, the capacity to use and adapt to uncertain contexts. Graduates have to act in a world of value conflicts, information limitations, vast registers of risks, and radical uncertainty. All this, along with the ongoing possibility of staying within personal and group ‘bubbles’ of and being exposed to vast operations of manipulation require a new thinking about the use of technology in education and a new set of graduate attributes. As advanced as AI solutions may become we cannot yet envisage a future where algorithms can really replace the complexity of human mind. For certain, current developments show that it is highly unlikely to happen in the next decade, despite a shared excessive optimism. The AI hype is not yet double by results; for example, Ruchir Puri, the Chief Architect of Watson, IBM’s AI supercomputer, recently noted that “There is a lot of hype around AI, but what it can’t do is very big right now. What it can do is very small.”

This reality may encourage policy-makers and experts to reimagine institutions of higher education in an entirely new paradigm, much more focused on imagination, creativity, and civic engagement. With the capacity to guide learning, monitor participation, and student engagement with the content, AI can customize the ‘feed’ of information and materials into the course according to learner’s needs, provide feedback and encouragement. However, teachers can use this to prepare students to a world of hyper-complexity where the future is not reduced to the simple aim of ‘employability.’ Teacherbots are already presenting as a disruptive alternative to traditional teaching staff, but it is very important to inquire at this point how do we use them for the benefit of students in the context of a profound rethink of what is currently labeled as ‘graduate attributes’ (Mason et al. 2016).

Even if in 2017 we find little and exploration of what is a teacherbot and what their capabilities are possible now and in a predictable future, AI technology has slipped into the backdoor of all our lives and this is imposing a much more focused research in higher education. AI solutions are currently monitoring our choices, preferences, movements, measuring strengths, and weaknesses, providing feedback, encouragement, badges, comparative analytics, customized news feeds, alerts, predictive text, so they are project managing our lives. At this point, we can see a teacherbot as a complex algorithmic interface able to use artificial intelligence for personalized education, able to provide bespoke content, supervision, and guidance for students and help for teachers. Teacherbots are defined as any machine-based software or hardware that assumes the role traditionally performed by a teacher assistant in organizing information and providing fast answers to a wide set of predictable questions; it can be facilitating, monitoring, assessing, and managing student learning within the online learning space. These solutions are closer than many academics may think. Tinkering with the old system of transmitting information to passive students, in class or in front of computers, is open to disruption from a highly personalized, scaleable, and affordable alternative AI solutions, such as ‘Jill Watson.’ While contact time and personal guidance by faculty may be should be retained not only in some elite institutions of higher education, as this will define the quality of education, but intelligent machines can be used by all to meet the learning and support needs of massive numbers of students.

## Conclusion

The rise of AI makes it impossible to ignore a serious debate about its future role of teaching and learning in higher education and what type of choices universities will make in regard to this issue. The fast pace of technology innovation and the associated job displacement, acknowledged widely by experts in the field (source), implies that teaching in higher education requires a reconsideration of teachers’ role and pedagogies. The current use of technological solutions such as ‘learning management systems’ or IT solutions to detect plagiarism already raise the question of who sets the agenda for teaching and learning: corporate ventures or institutions of higher education? The rise of techlords and the quasi-monopoly of few tech giants also come with questions regarding the importance of privacy and the possibility of a dystopian future. These issues deserve a special attention as universities should include this set of risks when thinking about a sustainable future.

Moreover, many sets of tasks that are currently placed at the core of teaching practice in higher education will be replaced by AI software based on complex algorithms designed by programmers that can transmit their own biases or agendas in operating systems. An ongoing critique and inquiry in proposed solutions stay critical to guarantee that universities remain institutions able to maintain civilization, promote, and develop knowledge and wisdom.

In effect, now is the time for universities to rethink their function and pedagogical models and their future relation with AI solutions and their owners. Furthermore, institutions of higher education see ahead the vast register of possibilities and challenges opened by the opportunity to embrace AI in teaching and learning. These solutions present new openings for education for all, while fostering lifelong learning in a strengthened model that can preserve the integrity of core values and the purpose of higher education.

We consider that there is a need for research on the ethical implications of the current control on developments of AI and the possibility to wither the richness of human knowledge and perspectives with the monopoly of few entities. We also believe that it is important to focus further research on the new roles of teachers on new learning pathways for higher degree students, with a new set of graduate attributes, with a focus on imagination, creativity, and innovation; the set of abilities and skills that can hardly be ever replicated by machines.
